# Pediatric screening urinalysis: a difference-in-differences analysis of how a 2007 change in guidelines impacted use

**DOI:** 10.1186/1471-2431-14-260

**Published:** 2014-10-10

**Authors:** Clara E Filice, Jeremy C Green, Marjorie S Rosenthal, Joseph S Ross

**Affiliations:** Robert Wood Johnson Foundation Clinical Scholars Program, Yale School of Medicine, 333 Cedar Street, SHM IE-61, PO Box 208088, New Haven, CT 06520 USA; Department of Health Management and Policy, College for Public Health and Social Justice, Saint Louis University, Saint Louis, MO USA; Department of Pediatrics, Yale School of Medicine, New Haven, CT USA; Department of Internal Medicine, Yale School of Medicine, New Haven, CT USA

**Keywords:** Preventive services, Practice guidelines, Screening urinalysis

## Abstract

**Background:**

Practice guidelines can promote higher-quality care, yet they are inconsistently adopted. The purpose of this study is to evaluate the impact of a 2007 American Academy of Pediatrics recommendation to discontinue routine screening urinalysis in children.

**Methods:**

Using data from the National Ambulatory Medical Care Survey, we used a difference-in-differences approach to estimate visit-level screening urinalysis proportions before (2005-2006, n = 1,247) and after (2008-2009, n = 1,772) the 2007 AAP recommendation. We compared visits by children 4-18 years old to visits by young adults aged 19-32. Analyses were adjusted for continuous patient age, patient race/ethnicity, physician specialty, and stratified by patient gender and visit setting.

**Results:**

The 2007 recommendation was associated with no significant change in adjusted visit-level screening urinalysis proportions in child visits (20.4% to 22.5%) compared to an increase in young adult visits (20.1% to 27.0%) – a differential impact of -4.8 percentage points (95% Confidence Interval [CI] -9.0, -0.5). In private practices, visit proportions differentially decreased by 7.6 percentage points (95% CI -13.7, -1.5) in female children and by 0.5 percentage points (95% CI -10.6, 9.6) in male children. In community health centers, visit proportions differentially decreased by 17.4 percentage points (95% CI -27.9, -6.8) in female children and by 33.5 percentage points (95% CI -47.4, -19.7) in male children.

**Conclusions:**

A 2007 recommendation to discontinue routine screening urinalysis in children was associated with no change in use in child visits relative to an increase in use in adult visits. Overall, nearly one-quarter of child visits still included screening urinalysis.

## Background

Standardized practice guidelines developed by professional societies and other health entities can help physicians make informed decisions about appropriate use of preventive services. Yet physicians who care for children, like many physicians, inconsistently utilize preventive care guidelines [1–3]. Attention to evaluating the quality of pediatric preventive care has been growing, and many previous studies [4–8] have examined whether recommended services are being delivered to children. Less attention has been devoted to evaluating whether services that are no longer recommended are being discontinued.

Recommendations regarding routine urine screening for the detection of renal or urologic disease in children have evolved over several decades. In both 1977 and 1991, the American Academy of Pediatrics (AAP) recommended routine urine screening at four time points during childhood [9]. Revised health supervision guidelines in 1995 [10] and 2000 [11] limited screening to only two age groups, five-year-olds and sexually active adolescents. Accumulated evidence now suggests that although inexpensive, screening urinalysis is a poor screening test for disease, it can lead to false positives and associated costly and invasive diagnostic evaluations, and there is limited evidence to suggest detection of abnormalities in childhood improves long-term outcomes [12–14]. In light of the lack of clear benefit associated with screening urinalysis relative to its associated costs and risks, the AAP in 2007 removed routine screening urinalysis for asymptomatic children and adolescents from its health supervision guidelines altogether [15]. In 2005, a survey of pediatricians showed that many still reported routinely screening children in non-recommended age groups [9]. Little is known about how this recent change in recommendations has impacted physician practice.

Using data from the National Ambulatory Medical Care Survey (NAMCS), a nationally representative survey assessing provision of ambulatory medical care services in the United States, we used a difference-in-differences approach to determine whether the 2007 AAP recommendation resulted in a differential impact on the proportion of child visits that included screening urinalysis compared to young adult visits. Because visit setting and patient gender may have an impact on screening urinalysis we stratified our analysis by visit setting (community health center or private practice) and by patient gender. Our findings will inform clinicians and guideline developers about current screening urinalysis practices among children, whether and how the AAP recommendation impacted physician behavior, and whether recommendations alone are sufficient in changing physician behavior.

## Methods

### Data source

We analyzed data from the National Ambulatory Medical Care Survey (NAMCS), a nationally representative data set of ambulatory visits to office-based physicians in the United States. Physicians, office staff, or survey administrators from the U.S. Bureau of the Census enter data, including demographic and clinical parameters, for a systematic random sample of visits; each visit is weighted to allow for extrapolation to nationally representative estimates. Conducted on an annual basis, the NAMCS offers a unique opportunity to estimate pediatric screening urinalysis prevalence before and after enactment of the 2007 AAP recommendation. Our study period of interest included two years before and two years after the AAP recommendation in 2007: from 2005-2006 and from 2008-2009. Data from 2007 were excluded to allow for an implementation period. The study was determined to be exempt from Committee Review by the Yale University Human Investigation Committee. The research has adhered to the STROBE guidelines for observational studies as outlined at http://www.strobe-statement.org.

### Study design and sample

We used a difference-in-differences approach [16] to quantify the impact of the AAP’s 2007 recommendation on the proportion of pediatric visits that included screening urinalysis. The study design involves consideration of study and comparison groups, before and after an intervention is applied to only the study group, in order to help identify differences in an outcome associated with the intervention separately from differences in covariates and secular trends (i.e. trends in testing over time that are unrelated to the timing of the AAP recommendation change and time-invariant differences between the two age groups).

Our study sample included visits to physicians who commonly see children, defined for the purpose of this analysis as pediatricians and family practice physicians. Visits to internal medicine physicians were excluded because it was anticipated that they would care for only adult populations. Because the NAMCS survey instrument does not distinguish between screening and diagnostic urinalysis, we defined screening urinalysis as urinalysis ordered in the context of a preventive care visit as designated by the physician; for each surveyed visit, providers are asked to select a categorical reason for the patient’s visit (new problem, chronic problem-routine, chronic problem-flare up, pre/post surgery, or preventive care). We studied children for whom catheterization would not likely be necessary to perform a screening urinalysis, defined for this analysis as those over age 4 years. Therefore, our analysis included visits by children aged 4-18 years.

For comparison, we designated a sample of visits to represent secular trends in screening behavior. Given our interest in the AAP recommendation, an ideal comparison group would be visits by children aged 4-18 years to physicians who were not exposed to the AAP recommendation. Due to the national applicability of the AAP guidance, data for such a comparison group was not available for visits in the United States. Rather, we selected as a comparison group preventive care visits to pediatricians or family practice physicians by young adults aged 19-32 years during the study periods of interest. To our knowledge universal screening urinalysis for adults was not recommended at any point in the study period, so its pattern of use was expected to reflect secular trends in physician screening practices and was not anticipated to be affected by the AAP guidance.

Our primary outcome of interest was the proportion of visits including screening urinalysis, defined as urinalysis ordered in the context of a physician-identified preventive care visit.

### Statistical analysis

We used NAMCS data to establish a baseline description of pediatric and young adult preventive care visits to pediatricians and family practitioners in 2005-2006, comparing unadjusted visit-level urinalysis proportions, patient gender, physician specialty, and visit setting. Our main analysis compared overall differences for visit-level rates of screening urinalysis in children before and after the 2007 AAP recommendation to the differences in young adults. We fit a nonlinear difference-in-differences model [16], adjusting for covariates, age category, and time period using probit regression.

Analyses were adjusted for or stratified by patient- and physician-level characteristics we anticipated might impact screening urinalysis use. We adjusted for the patient-level variable of continuous age because we anticipated it could affect prevalence of testing, given previous age-specific recommendations; analyses were also adjusted for patient race/ethnicity (collapsed into three categories, white, black, or other race/ethnicity because of small sample sizes in other categories). We also adjusted for physician specialty (pediatrician or family practitioner,) because we anticipated pediatricians may be differentially receptive to guidance from a pediatric-specific professional organization. Sample size limitations precluded a stratified analysis by physician specialty type. We anticipated practice setting may have an impact on screening urinalysis use due to factors such as laboratory access, patient insurance mix, and provider characteristics, so analyses were stratified by the system-level variable of visit setting (private practice or community health center). Because screening urinalysis may be differentially utilized between females (for purposes such as pregnancy testing) and males, we also stratified analysis by patient gender. Data from our NAMCS sample were weighted according to the National Center for Health Statistics weighting procedure to produce national estimates and adjusted for the survey design [17]. Computation was in Stata version 12 [18].

## Results

### Baseline characteristics

This analysis included preventive care visits from two time periods (2005-2006 and 2008-2009) involving two age groups (children 4-18 years and young adults 19-32 years). Overall, we analyzed 3,019 preventive care visits: 1,247 visits from 2005-2006 and 1,772 visits from 2008-2009. Before 2007, a majority of child and adult visits occurred in the private practice setting (95.6% and 97.3%, respectively). The majority of visits by children (75.7%) were to pediatricians while most visits by young adults (93.7%) were to family medicine practitioners; young adult visits were predominantly made by females (71.1%) (Table 1).

**Table 1:** **Baseline characteristics of preventive care visits before 2007 (2005-2006)**

	Child visits				Young adult visits			
	Unweighted ^a^ (N = 916)		Weighted ^b^ (N = 36,247,982)		Unweighted (N = 331)		Weighted (N = 10,693,301)	
Characteristic	Visits (N)	Proportion of visits (%)	Visits (N)	Proportion of visits (%, (95% CI ^c^))	Visits (N)	Proportion of visits (%)	Visits (N)	Proportion of visits (%, (95% CI))
**Urinalysis**	180	19.7	7,347,141	20.3 (15.0, 26.8)	63	19.0	2,158,310	20.2 (14.6, 27.2)
**Patient gender**								
Male	473	51.64	19,231,738	53.1 (47.9, 58.2)	85	25.7	3,087,855	28.9 (21.8, 37.1)
Female	443	48.36	17,016,244	46.9 (41.9, 52.1)	246	74.3	7,605,446	71.1 (62.9, 78.2)
**Patient race/ethnicity**								
White	694	75.8	30,532,293	84.2 (78.7, 88.6)	240	72.5	8,441,612	78.9 (70.1, 85.7)
Black	119	13.0	3,068,705	8.5 (5.4, 13.0)	47	14.2	1,397,124	13.1 (8.3, 20.0)
Other	103	11.2	2,646,984	7.3 (4.9, 10.7)	44	13.3	854,565	8.0 (4.0, 15.3)
**Physician specialty**								
Pediatrician	660	72.1	27,449,990	75.7 (69.3, 81.2)	14	4.2	679,551	6.4 (2.7, 14.1)
Family practitioner	256	28.0	8,797,992	24.3 (18.8, 30.7)	317	95.8	10,013,750	93.7 (85.9, 97.3)
**Visit setting**								
Private practice	686	74.9	34,641,012	95.6 (91.8, 97.7)	231	69.8	10,406,117	97.3 (94.8, 98.6)
CHC	230	25.1	1,606,970	4.4 (2.4, 8.2)	100	30.2	287,184	2.7 (1.4, 5.2)

^a^Unweighted estimates reflect the absolute number of visits in the study sample.

^b^Weighted estimates are sample visits weighted using NAMCS patient visit frequencies to develop national estimates.

^c^CI = Confidence Interval.

### Screening urinalysis before and after the 2007 AAP policy statement

Overall, the adjusted proportion of visits including screening urinalysis prior to the 2007 AAP policy statement were similar in preventive care visits by children aged 4-18 years and visits by young adults aged 19-32 years. Adjusted screening urinalysis proportions before and after the 2007 recommendation remained flat in child visits, from 20.4% before to 22.5% after, but increased in young adult visits, from 20.1% to 27.0%, resulting in a differential impact on child visits of -4.8 percentage points (95% confidence interval [CI], -9.0 to -0.5, p = 0.03).

Compared to young adults, after stratifying analyses by visit setting and patient gender, adjusted screening urinalysis proportions in female child visits to private practices differentially decreased by 7.6 percentage points (95% CI -13.7 to -1.5, p = 0.02). In male child visits to private practices, proportions differentially decreased by 0.5 percentage points (95% CI -10.6 to 9.6, p = 0.93). In community health center visits by female children, proportions differentially decreased by 17.4 percentage points (95% CI -27.9 to -6.8, p = 0.001); in community health center visits by male children, proportions differentially decreased by 33.5 percentage points (95% CI -47.4 to -19.7, p < 0.001) (Table 2). Of note, sample sizes were small for some cells, leading to imprecise estimates.

**Table 2:** **Adjusted proportion of child visits including urinalysis before and after 2007, compared to young adult visits**

Visit sample	Total unweighted visits (N) ^a^	Total weighted visits (N) ^b^	Before, 2005-2006 proportion of visits, % (95% CI ^c^) ^d^	After, 2008-2009 proportion of visits, % (95% CI) ^d^	Before-after difference,% (95% CI)	Difference-in-differences, % (95% CI)	P-value
**Overall**							
Females and males							
Children	2,151	80,088,667	20.4 (18.4, 22.5)	22.5 (21.9, 23.2)	2.1 (-0.3, 4.5)	-4.8 (-9.0, -0.5)	0.03
Young adults	868	23,244,921	20.1 (14.7, 25.5)	27.0 (23.7, 30.3)	6.9 (0.6, 13.2)		
**Private practices**							
Females							
Children	773	36,124,192	23.3 (14.6, 31.9)	22.5 (20.3, 24.7)	-0.7 (-9.8, 8.4)	-7.6 (-13.7, -1.5)	0.02
Young adults	379	15,179,624	20.4 (14.9, 25.9)	27.3 (24.9, 29.6)	6.9 (1.1, 12.7)		
Males							
Children	875	40,420,271	18.3 (15.4, 21.2)	23.6 (21.4, 25.9)	5.4 (2.2, 8.5)	-0.5 (-10.6, 9.6)	0.93
Young adults	149	6,024,527	18.2 (11.8, 24.7)	24.1 (16.5, 31.6)	5.8 (-3.8, 15.5)		
**Community health centers**							
Females							
Children	271	1,823,073	29.2 (27.4, 30.9)	6.2 (3.3, 9.1)	-23.0 (-27.5, -18.4)	-17.4 (-27.9, -6.8)	0.001
Young adults	302	1,827,843	34.9 (31.8, 38.0)	29.3 (24.1, 34.5)	-5.6 (-12.8, 1.6)		
Males							
Children	232	1,721,131	13.7 (4.6, 22.9)	5.6 (2.0, 9.1)	-8.2 (-18.1, 1.8)	-33.5 (-47.4, -19.7)	<0.001
Young adults	47	212,927	10.6 (10.2, 11.0)	36.0 (18.0, 54.0)	25.3 (7.7, 43.0)		

^a^Unweighted visits reflect the absolute number of visits in the study sample.

^b^Weighted visits are sample visits weighted using NAMCS patient visit frequencies to develop national estimates.

^c^CI = Confidence Interval.

^d^Estimates are adjusted for continuous patient age, patient race/ethnicity (white, black, and other), and physician specialty (pediatrician or family practitioner).

## Discussion

In the two year periods before and after a 2007 recommendation to discontinue routine screening urinalysis in children, we observed no change in screening urinalysis use in child visits. In adult visits, screening urinalysis rates rose, resulting in a differential decrease in child visits of nearly 5 percentage points. Our stratified analyses by visit setting and patient gender also revealed differential impacts in child visits compared to young adult visits. Overall, screening urinalysis use persisted in nearly a quarter of pediatric preventive care visits after 2007.

The increase in urinalyses observed in adult visits could be explained by several factors. A concurrent change in adult guidelines could lead to an increase in the proportion of adult visits including screening urinalysis. However, at no time during the study period was universal screening urinalysis recommended by major adult preventive care guidelines developers such as the United States Preventive Services Task Force (USPSTF) [19, 20] or the American Academy of Family Physicians (AAFP) [21, 22], making this explanation less likely. Or, it could be attributed to an increase in adult visits by subgroups for whom urinalysis may be an appropriate screening test, such as pregnant women or patients with diabetes, kidney disease, or hypertension. Similarly, routine urine chlamydia tests could have been misclassified as urinalyses. However, we did not anticipate significant differences in the proportions of these subgroups visiting physicians in either age group in the short period before or after the AAP recommendation, so find this explanation to be less likely as well. Therefore, we interpret the absolute increase in the proportion of adult visits including screening urinalysis to a general upward trend in screening tests – a trend that could be accounted for in part by increased adoption of the medical record, which has been associated with increased provision of preventive health services generally [23, 24].

The observed differential decrease in pediatric screening urinalysis in our study was consistent with the observed impact of another recently discontinued preventive care practice. In 2008, the USPSTF recommended discontinuation in older men of prostate cancer screening with the prostate-specific antigen test, which was shown to result in a small but significant impact on screening [25]. Unfortunately, discontinued routine child preventive care screening tests are not common, so comparison in the pediatric population is difficult.

Our stratified analyses by visit setting and patient gender also revealed differential impacts. We observed consistently larger differential decreases in screening urinalysis proportions in the community health center setting than in the private practice setting for both genders. This finding is consistent with previous studies demonstrating comparable or higher quality care delivery in community health center settings compared to other settings [26–28]. Community health centers have also been shown to provide cost-effective care [28], which could make it more likely that community health center practitioners would more rapidly discontinue a procedure such as urinalysis (which can be time-intensive and minimally revenue-generating) once it was no longer recommended. Our gender-stratified results were mixed and thus more difficult to interpret; while urinalysis in female child visits differentially decreased in both settings, in male visits we observed a large differential decrease in the community health center setting compared to no change in the private practice setting.

Despite observed differential decreases, the overall proportion of pediatric visits including screening urinalysis remained high. The persistence of screening urinalysis use – in nearly a quarter of all child visits - could be explained by a physician lack of awareness of the AAP guideline [1–3]. Additionally, physicians are subject to a wide array of clinical directives – a 2006 study [29] identified over 340 AAP policy statements alone, of which 57 were deemed broadly relevant to pediatric practice. Alternatively, physicians may have experienced previously cited barriers to guideline adoption, such as lack of awareness or familiarity, lack of self-efficacy in adopting a guideline, or inability to overcome the inertia of previous practice [30], many of which have been noted in studies of pediatricians and family practitioners [1–3, 31]. Another possibility is that physicians were considering other, potentially conflicting, input when making decisions about the use of screening urinalysis. The appropriate use of screening urinalysis as a routine screening test has been debated for decades and the discourse continues [32]. However, in this case, a relatively high degree of consensus regarding pediatric screening urinalysis had been reached with concordant guidelines from at least several major guideline developers, including the USPSTF [19, 20], the AAFP [21, 22], and the American Medical Association’s Guidelines for Adolescent Preventive Services [33]. Physicians may also have conducted screening urinalyses for other reasons than to detect disease, for instance to meet requirements for school entry or sports participation that had not been changed to reflect new evidence about the utility of the screening test [34].

This study’s strengths include use of a strong, quasi-experimental, difference-in-differences approach [35] to evaluate the impact of the 2007 AAP recommendation. Our research design accounts for secular time trends in test use and differential time trends in unobserved covariates between the age groups. Furthermore, it did not depend on physician report of screening practices to measure screening urinalysis rates. However, it is important to consider the limitations of this analysis as well. Because the NAMCS visit form does not distinguish between screening and diagnostic urinalyses, our sample could include visits by patients who presented for a preventive care visit who simultaneously had acute complaints that would warrant a diagnostic urinalysis – leading to misclassification. There are a number of other conditions and circumstances in which urinalyses would be warranted in the context of preventive care visits, noted above, that could have biased our results. Additionally, this sample includes only physicians; patterns of adherence may be different for other types of practitioners.

## Conclusions

In conclusion, our study offers evidence that a 2007 standardized practice guideline differentially impacted routine screening urinalysis use in child compared to adult ambulatory care visits. Differential impacts were noted across visit settings and patient genders. However, screening urinalysis use persisted in nearly a quarter of pediatric preventive care visits. More research is needed to understand why and how females and males are impacted differentially by screening guidelines, what differentiates practice settings, and why physicians do or do not discontinue screening tests once they are no longer recommended.

## Abbreviations

AAP: American Academy of Pediatrics
AAFP: American Academy of Family Physicians
CI: Confidence interval
NAMCS: National Ambulatory Medical Care survey
USPSTF: United States Preventive Services Task Force.
